# Independent Principal Component Analysis for biologically meaningful dimension reduction of large biological data sets

**DOI:** 10.1186/1471-2105-13-24

**Published:** 2012-02-03

**Authors:** Fangzhou Yao, Jeff Coquery, Kim-Anh Lê Cao

**Affiliations:** 1 Shanghai University of Finance and Economics, Shanghai, P.R. China; 2 Queensland Facility for Advanced Bioinformatics, University of Queensland, St Lucia, QLD 4072, Australia; 3 Sup'Biotech, Villejuif, F-94800, France

## Abstract

**Background:**

A key question when analyzing high throughput data is whether the information provided by the measured biological entities (gene, metabolite expression for example) is related to the experimental conditions, or, rather, to some interfering signals, such as experimental bias or artefacts. Visualization tools are therefore useful to better understand the underlying structure of the data in a 'blind' (unsupervised) way. A well-established technique to do so is Principal Component Analysis (PCA). PCA is particularly powerful if the biological question is related to the highest variance. Independent Component Analysis (ICA) has been proposed as an alternative to PCA as it optimizes an independence condition to give more meaningful components. However, neither PCA nor ICA can overcome both the high dimensionality and noisy characteristics of biological data.

**Results:**

We propose Independent Principal Component Analysis (IPCA) that combines the advantages of both PCA and ICA. It uses ICA as a denoising process of the loading vectors produced by PCA to better highlight the important biological entities and reveal insightful patterns in the data. The result is a better clustering of the biological samples on graphical representations. In addition, a sparse version is proposed that performs an internal variable selection to identify biologically relevant features (sIPCA).

**Conclusions:**

On simulation studies and real data sets, we showed that IPCA offers a better visualization of the data than ICA and with a smaller number of components than PCA. Furthermore, a preliminary investigation of the list of genes selected with sIPCA demonstrate that the approach is well able to highlight relevant genes in the data with respect to the biological experiment.

IPCA and sIPCA are both implemented in the R package mixomics dedicated to the analysis and exploration of high dimensional biological data sets, and on mixomics' web-interface.

## Background

With the development of high throughput technologies, such as microarray and next generation sequencing data, the exploration of high throughput data sets is becoming a necessity to unveil the relevant information contained in the data. Efficient exploratory tools are therefore needed, not only to assess the quality of the data, but also to give a comprehensive overview of the system, extract significant information and cope with the high dimensionality. Indeed, many statistical approaches fail or perform poorly for two main reasons: the number of samples (or observations) is much smaller than the number of variables (the biological entities that are measured) and the data are extremely noisy.

In this study, we are interested in the application of unsupervised approaches to discover novel biological mechanisms and reveal insightful patterns while reducing the dimension in the data. Amongst the different categories of unsupervised approaches (clustering, model-based and projection methods), we are specifically interested in projection-based methods, which linearly decompose the data into components with a desired property. These exploratory approaches project the data into a new subspace spanned by the components. They allow dimension reduction without loss of essential information and visualization of the data in a smaller subspace.

Principal component analysis (PCA) [1] is a classical tool to reduce the dimension of expression data, to visualize the similarities between the biological samples, and to filter noise. It is often used as a pre-processing step for subsequent analyses. PCA projects the data into a new space spanned by the principal components (PC), which are uncorrelated and orthogonal. The PCs can successfully extract relevant information in the data. Through sample and variable representations, they can reveal experimental characteristics, as well as artefacts or bias. Sometimes, however, PCA can fail to accurately reflect our knowledge of biology for the following reasons: a) PCA assumes that gene expression follows a multivariate normal distribution and recent studies have demonstrated that microarray gene expression measurements follow instead a super-Gaussian distribution [2-5], b) PCA decomposes the data based on the maximization of its variance. In some cases, the biological question may not be related to the highest variance in the data [6].

A more plausible assumption of the underlying distribution of high-throughput biological data is that feature measurements following Gaussian distributions represent noise - most genes conform to this distribution as they are not expected to change at a given physiological or pathological transition [7]. Recently, an alternative approach called Independent Component Analysis (ICA) [8-10] has been introduced to analyze microrray and metabolomics data [2,6,11-13]. In contrary to PCA, ICA identifies non-Gaussian components which are modelled as a linear combination of the biological features. These components are statistically independent, i.e. there is no overlapping information between the components. ICA therefore involves high order statistics, while PCA constrains the components to be mutually orthogonal, which involves second order statistics [14]. As a result, PCA and ICA often choose different subspaces where the data are projected. As ICA is a blind source signal separation, it is used to reduce the effects of noise or artefacts of the signal since usually, noise is generated from independent sources [10]. In the recent literature, it has been shown that the independent components from ICA were better at separating different biological groups than the principal components from PCA [2,5-7]. However, although ICA has been found to be a successful alternative to PCA, it faces some limitations due to some instability, the choice of number of components to extract and high dimensionality. As ICA is a stochastic algorithm, it needs to be run several times and the results averaged in order to obtain robust results [5]. The number of independent component to extract and choose is a hard outstanding problem. It has been the convention to use a fixed number of components [2]. However, ICA does not order its components by 'relevance'. Therefore, some authors proposed to order them either with respect to their kurtosis values [9], or with respect to their l_2 _norm [2], or by using Bayesian frameworks to select the number of components [15]. In the case of high dimensional data sets, PCA is often applied as a pre-processing step to reduce the number of dimensions [2,7]. In that particular case, ICA is applied on a subset of data summarized by a small number of principal components from PCA.

In this paper, we propose to use ICA as a denoising process of PCA, since ICA is good at separating mixed signals, i.e. noise vs. no noise. The aim is to generate denoised loading vectors. These vectors are crucial in PCA or ICA as each of them indicates the weights assigned to each biological feature in the linear combination that leads to the component. Therefore, the goal is to obtain independent components that better reflect the underlying biology in a study and achieve better dimension reduction than PCA or ICA.

Independent Principal Component Analysis (IPCA) makes the assumption that biologically meaningful components can be obtained if most noise has been removed in the associated loading vectors.

In IPCA, PCA is used as a pre-processing step to reduce the dimension of the data and to generate the loading vectors. The FastICA algorithm [9] is then applied on the previously obtained PCA loading vectors that will subsequently generate the Independent Principal Components (IPC). We use the kurtosis measure of the loading vectors to order the IPCs. We also propose a sparse variant with a built-in variable selection procedure by applying soft-thresholding on the independent loading vectors [16,17] (sIPCA).

In the 'Results and Discussion' Section, we first compare the classical PCA and ICA methodologies to IPCA on a simulation study. On three real biological datasets (microarray and metabolomics datasets) we demonstrate the satisfying samples clustering abilities of IPCA. We then illustrate the usefulness of variable selection with sIPCA and compare it with the results obtained from the sparse PCA from [18]. In the 'Methods' Section, we present the PCA, ICA and IPCA methodologies and describe how to perform variable selection with sIPCA.

## Results and Discussion

We first performed a simulation study where the loading vectors follow a Gaussian or super-Gaussian distribution. On three real data sets, we compared the kurtosis values of the loading vectors as a way of measuring their non-Gaussianity and ordering the IPCs. The samples clustering ability of each approach is assessed using the Davies Bouldin index [19]. Finally, the variable selection performed by sIPCA and sPCA are compared on a simulated as well as on the Liver Toxicity data sets.

### Simulation study

In order to understand the benefits of IPCA compared to PCA or ICA, we simulated 5000 data sets of size *n *= 50 samples and *p *= 500 variables from a multivariate normal distribution with a pre-specified variance-covariance matrix described in the 'Methods' Section. Two cases were tested.

1. Gaussian case. The first two eigenvectors **v**_1 _and **v**_2_, both of length 500, follow a Gaussian distribution.

2. Super-Gaussian case. In this case the first two eigenvectors follow a mixture of Laplacian and uniform distributions:

$$v_{1k}\sim \left\{\begin{array}{cc} L\left(0,25\right) & k=1,\ldots ,50\\ U\left(0,1\right) & \text{otherwise}, \end{array}\right)\;\;\text{and}\;\;v_{2k}\sim \left\{\begin{array}{cc} L\left(0,25\right) & k=301,\ldots ,350\\ U\left(0,1\right) & \text{otherwise}. \end{array}\right)$$

Table 1 records the median of the angles between the simulated (known) eigenvectors and the loading vectors estimated by the three approaches. PCA gave similar results in both simulation cases, and was able to well estimate the loading vectors, while ICA performed poorly in both cases. IPCA performed quite poorly in the Gaussian case, but outperformed PCA in the super-Gaussian case.

**Table 1:** Simulation study: angle (median value) between the simulated and estimated loading vectors simulated with either Gaussian or super-Gaussian distributions.

Method	Gaussian		super-Gaussian	
	
	v_1_	v_2_	v_1_	v_2_
PCA	20.48	21.61	20.47	21.62
ICA	85.70	84.39	82.13	77.77
IPCA	70.05	69.72	12.46	14.08

Table 2 displays the kurtosis values of the first 5 loading vectors. In IPCA the components are ordered with respect to the kurtosis values of their associated loading vectors, while in the FastICA algorithm the components are ordered with respect to the kurtosis values of the independent components. In the super-Gaussian case, these results show that the kurtosis value is a good post hoc indicator of the number of components to choose, as a sudden drop in the values corresponds to irrelevant dimensions (from 3 and onwards). Low kurtosis values in the Gaussian case indicate that non-Gaussianity of the loading vectors cannot be maximized, and that the assumptions of IPCA are not met (i.e. a small number of genes heavily contribute to the observed biological process).

**Table 2:** Mean value of the kurtosis measure of the first 5 loading vectors in the simulation study for PCA, IPCA and & ICA.

		PCA	ICA	IPCA
Gaussian case	loading 1	-0.007	-0.015	0.54
	loading 2	-0.009	-0.013	0.21
	loading 3	-0.012	-0.013	-0.01
	loading 4	-0.011	-0.013	-0.20
	loading 5	-0.015	-0.015	-0.41

super-Gaussian case	loading 1	34.75	0.28	52.58
	loading 2	34.16	0.43	33.81
	loading 3	-0.01	0.42	0.27
	loading 4	-0.01	0.44	-0.02
	loading 5	-0.02	0.47	-0.25

Tables 1 and 2 seem to suggest that ICA performs poorly in both Gaussian and super-Gaussian case, even if we would expect quite the contrary in the super-Gaussian case. In the high dimensional case, PCA is used as a pre processing step in the ICA algorithm. It is likely that such step affects the ICA input matrix and that the ICA assumptions are not met. Therefore, the performance of ICA seems to be largely affected by the high number of variables.

PCA gave satisfactory results in both cases. In the super-Gaussian case, PCA is even able to recover some of the super-Gaussian distribution of the loading vectors. However, IPCA is able to recover the loading structure better than PCA in the super-Gaussian case (angles are smaller in Table 1 and kurtosis value is much higher for the first loading for IPCA). Depending on the (unknown) nature of the data set to be analyzed, it is therefore advisable to assess both approaches.

### Application to real data sets

#### Liver Toxicity study

In this study, 64 male rats were exposed to non-toxic (50 or 150 mg/kg), moderately toxic (1500 mg/kg) or severely toxic (2000 mg/kg) doses of acetaminophen (paracetamol) in a controlled experiment [20]. In this paper, we considered 50 and 150 mg/kg as low doses, and 1500 and 2000 as high doses. Necropsies were performed at 6, 18, 24 and 48 hours after exposure and the mRNA from the liver was extracted. The microarray data is arranged in matrix of 64 samples and 3116 transcripts.

#### Prostate cancer study

This study investigated whether gene expression differences could distinguish between common clinical and pathological features of prostate cancer. Expression profiles were derived from 52 prostate tumors and from 50 non tumor prostate samples (referred to as normal) using oligonucleotide microarrays containing probes for approximately 12,600 genes and ESTs. After preprocessing remains the expression of 6033 genes (see [21]) and 101 samples since one normal sample was suspected to be an outlier and was removed from the analysis.

#### Yeast metabolomic study

In this study, two Saccharomyces cerevisiae strains were used - wild-type (WT) and mutant (MT), and were carried out in batch cultures under two different environmental conditions, aerobic (AER) and anaerobic (ANA) in standard mineral media with glucose as the sole carbon source. After normalization and preprocessing, the metabolomic data results in 37 metabolites and 55 samples that include 13 MT-AER, 14 MT-ANA, 15 WT-AER and 13 WT-ANA samples (see [22] for more details).

#### Choosing the number the components with the kurtosis measure

As mentioned by [5], one major limitation of ICA is the specification and the choice of the number of components to extract. In PCA, the cumulative percentage of explained variance is a popular criterion to choose the number of principal components, since they are ordered by decreasing explained variance [1]. For the case of high dimensionality, many alternative ad hoc stopping rules have been proposed without, however, leading to a consensus (see [23] for a thorough review). In Liver Toxicity, the first 3 principal components explained 63% of the total variance, in Yeast, the first 2 principal components explained 85% of the total variance. For Prostate that contains a very large number of variables, the first 3 components only explain 51% of the total variance (7 principal components would be necessary to explain more than 60%). However, from a visualization perspective, choosing more than 3 components would be difficult to interpret.

The kurtosis values of the loading vectors from PCA, ICA and IPCA are displayed in Table 3. These values differ from one approach to the others, as well as their order. In IPCA, the kurtosis value of the associated loading vectors gives a good indicator of the ability of the components to separate the clusters, since we are interested in extracting signals from non-Gaussian distributions. Respectively, the first 2, 1 and 2 components seem enough in Liver Toxicity, Prostate and Yeast to extract relevant information with IPCA, as is further discussed below.

**Table 3:** Kurtosis measures of the loading vectors for PCA, IPCA and & ICA.

Dataset		PCA	ICA	IPCA
Liver Toxicity study	loading 1	6.588	7.697	9.700
	loading 2	1.912	2.737	6.982
	loading 3	6.958	4.799	0.672

Prostate cancer study	loading 1	-1.527	-0.553	1.513
	loading 2	-0.561	0.723	-0.249
	loading 3	1.176	1.640	-1.509

Yeast metabolomic study	loading 1	4.532	0.274	1.551
	loading 2	12.261	-0.758	1.437
	loading 3	4.147	1.677	-0.475

#### Sample representation

**Figure 1:**
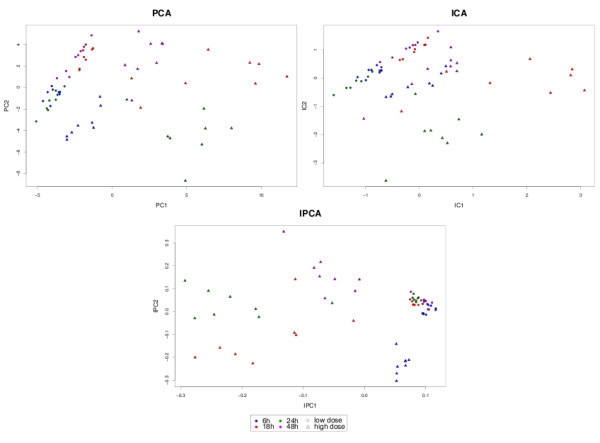
**Liver Toxicity study: Sample representation**. Sample representation using the first two components from PCA, ICA and IPCA approaches.

**Figure 2:**
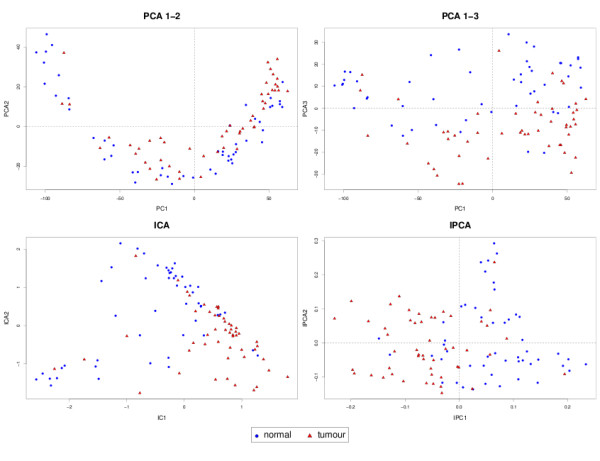
**Prostate cancer study: sample representation**. Sample representation using the first two or three components from PCA, ICA and IPCA approaches.

**Figure 3:**
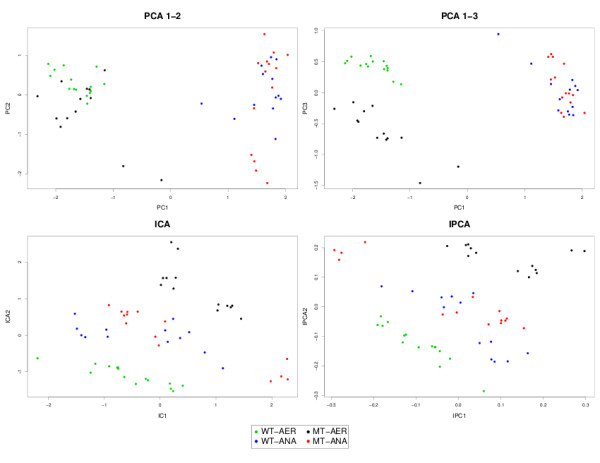
**Yeast metabolomic study: sample representation**. Sample representation using the first two or three components from PCA, ICA and IPCA approaches.

The samples in each data set were projected in the new subspace spanned by the PCA, ICA or IPCA components (Figure 1, 2 and 3). This kind of graphical output gives a better insight into the biological study as it reveals the shared similarities between samples. The comparison between the different graphics allows to visualize how each method is able to partition the samples in a way that reflects the internal structure of the data, and to extract the relevant information to represent each sample. One would expect that the samples belonging to the same biological group, or undergoing the same biological treatment would be clustered together and separated from the other groups.

In Liver Toxicity, IPCA tended to better cluster the low doses together, compared to PCA or ICA (Figure 1). In Prostate (Figure 2), PCA graphical representations showed interesting patterns. Neither the first, nor the second component in PCA were relevant to separate the two groups. Instead, it was the third component that could give more insight into the expected biological characteristics of the samples. It is likely that PCA first attempts to maximize the variance of noisy signals, which has a Gaussian distribution, before being able to find the right direction to differentiate better the sample classes. For IPCA, the first component seemed already sufficient to separate the classes (as indicated by the kurtosis value of its associated loading vector in Table 3), while two components were necessary for ICA to achieve a satisfying clustering. For the Yeast study (Figure 3), even though the first 2 principal components explained 85% of the total variance, it seemed that 3 components were necessary to separate WT from the MT in the AER samples with PCA, whereas 2 components were sufficient with ICA and IPCA. For all approaches, the WT and MT samples for the ANA group remain mixed and seem to share strong biological similarities.

#### Cluster validation

In order to compare how well different methods perform on a data set, different indexes were proposed to measure the similarities between clusters in the literature [24]. We used the Davies-Bouldin index [19] (see 'Methods' section). This index has both a statistical and geometric rationale, and looks for compact and well-separated clusters. The main purpose is to check whether the different approaches can distinguish between the known biological conditions or treatments on the basis of the expression data. The approach that gives the smallest index is considered the best clustering method based on this criterion. The results are displayed in Table 4 for a choice of 2 or 3 components. On the Liver Toxicity study, the Davies-Bouldin index indicated that IPCA outperformed the other approaches using 2 components. When choosing 3 components, all approaches gave similar results. On Prostate, ICA slightly outperformed IPCA for 2 components and gave similar performances for 3 components. PCA seemed clearly limited by the large number of noisy variables and was not able to provide a satisfying clustering of the samples. ICA gave good clustering performance on the Yeast data set for 2 components, followed by PCA and IPCA. It is probable that there is very little noise in this small data set.

**Table 4:** Davies Bouldin index for PCA, ICA and IPCA on the three data sets.

Dataset	# of components	PCA	ICA	IPCA
Liver Toxicity study	2 components	1.809	1.923	1.242
Liver Toxicity study	3 components	1.523	1.578	1.525

Prostate cancer study	2 components	4.117	1.679	1.782
Prostate cancer study	3 components	3.312	2.316	2.315

Yeast metabolomic study	2 components	1.894	1.788	2.338
Yeast metabolomic study	3 components	2.119	2.139	2.037

In fact, the Davies-Bouldin index seemed to indicate that for large data sets (Liver Toxicity and Prostate), IPCA seems to perform best for a smaller number of components than PCA. It is able to highlight relevant information in a very small number of dimensions.

#### Variable selection

We first performed a simulation study to assess whether sIPCA could identify relevant variables. We then applied sIPCA to the Liver Toxicity study. In both cases, we compared sIPCA with the sparse PCA approach (sPCA-rSVD-soft from [18]) that we will subsequently call 'sPCA'.

### Simulated example

Using the simulation framework described in the 'Methods' Section, we considered two cases:

1. Gaussian case. The two sparse simulated eigenvectors followed a Gaussian distribution:

$$v_{1k}\left\{\begin{array}{cc} \sim N\left(0,1\right) & k=1,\ldots ,50\\ =0 & \text{otherwise}, \end{array}\right)\;\;\text{and}\;\;v_{2k}\left\{\begin{array}{cc} N\left(0,1\right) & k=301,\ldots ,350\\ =0 & \text{otherwise}. \end{array}\right)$$

2. Super-Gaussian case. In this case, we have

$$v_{1k}\left\{\begin{array}{cc} \sim L\left(0,25\right) & k=1,\;\ldots ,\;50\\ =0 & \text{otherwise}, \end{array}\right)\;\;\text{and}\;\;v_{2k}\left\{\begin{array}{cc} \sim L\left(0,25\right) & k=301,\ldots ,350\\ =0 & \text{otherwise}. \end{array}\right)$$

Each eigenvector has 50 non-zero variables and the coefficients in the loading vectors associated to these non-zero variables follow a Gaussian or super-Gaussian distribution. sPCA and sIPCA were then applied on each generated data set. Both approaches require the degree of sparsity, which was set to 50, as an input parameter on each component. One can imagine that each eigenvector describes a particular biological process where 50 genes contribute heavily or very heavily to. Table 5 displays the correct identification rate of each loading vector estimated by sPCA and sIPCA. Given this non trivial setting, both approaches identified very well the important variables, especially on the first dimension, where sPCA slightly outperformed sIPCA. On the second dimension, the performance of sPCA and sIPCA differ as sPCA fails to differentiate each sparse signal separately - it tended to select variables from both dimensions in the second loading vector. On the contrary, and especially in the super-Gaussian case, sIPCA is able to identify each sparse eigenvector signal separately, i.e. each simulated biological process. sPCA performed better in the Gaussian than in the super-Gaussian case, whereas sIPCA performed almost equally well in both cases.

**Table 5:** Simulation study: average percentage of correctly identified non-zero loadings (standard deviation) when 50 variables are selected on each dimension (each loading vector).

Method	Gaussian		super-Gaussian	
	
	v_1_	v_2_	v_1_	v_2_
sPCA	90.30% (3.5)	72.5% (11.6)	85.44% (4.3)	68.22% (10.6)
sIPCA	86.7% (8.3)	87.7% (8.1)	80.80% (8.6)	82.30% (8.4)

### Real example with Liver Toxicity study

#### Choosing the number of genes to select

**Figure 4:**
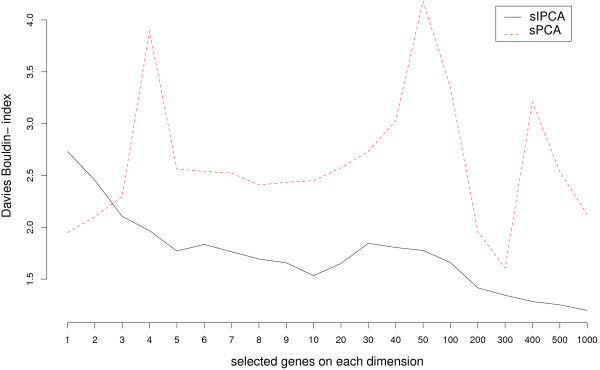
**Liver Toxicity study: Davis Bouldin index for sIPCA and sPCA**. Comparison of the Davies Bouldin index for sIPCA and sPCA with respect to the number of variables selected on 2 components.

Figure 4 displays the Davies Bouldin index for various gene selection sizes. sIPCA clearly outperformed sPCA. In order to compare the biological relevance of the two gene selections, a selection size of 50 genes per dimension, for 2 dimensions were arbitrarily chosen for the following analysis. Even if not optimal from the index perspective, this choice was mostly guided by the number of subsequent annotated genes that could be analyzed in the biological interpretation. For each approach, the genes lists of different sizes are embedded into each other, and a compromise has to be made to obtain a sufficient but not too large list of genes to be interpreted.

#### Comparison of the sparse loading vectors

**Figure 5:**
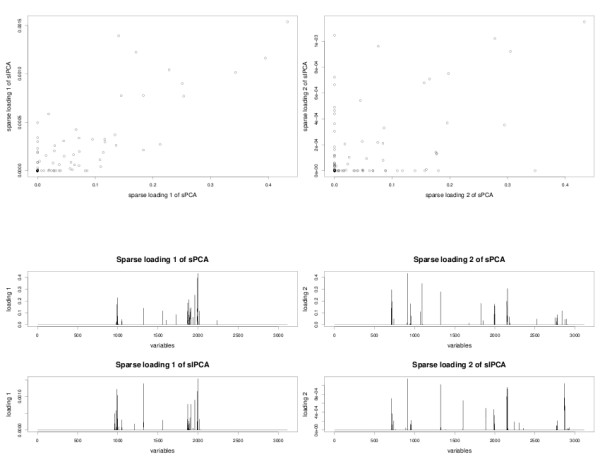
**Liver Toxicity study: sparse loading vectors**. Comparison of the first two sparse loading vectors generated by sIPCA and sPCA.

The first and second sparse loading vectors for both sPCA and sIPCA are plotted in Figure 5 (absolute values). In the first dimension, the loading vectors of the two sparse approaches are very similar (correlation of 0.98), a fact that was already indicated in the above simulation study. Both approaches select the same variables. On the second dimension, however, the sparse loading vectors differ (correlation of 0.28) as IPCA (similar to ICA) leads to an unnecessarily orthogonal basis which may reconstruct the data better than PCA in the presence of noise and is sensitive to high order statistics in the data rather than the covariance matrix only [25]. This explains why sPCA and sIPCA give different subspaces.

#### Sample representation

**Figure 6:**
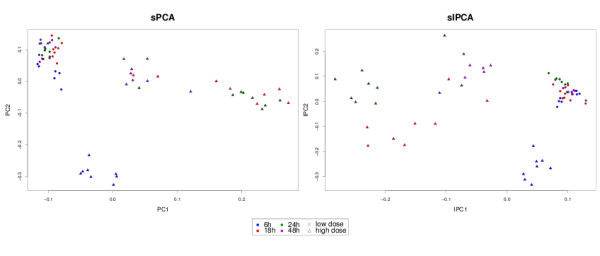
**Liver Toxicity study: sample representation with sparse variants**. Sample representation using the first two principal components of sPCA and sIPCA approaches when 50 variables are selected on each dimension.

The PCs and IPCs are displayed in Figure 6. Since most of the noisy variables were removed, sPCA seemed to give a better clustering of the low doses compared to Figure 1. sIPCA and IPCA remain similar, which shows that IPCA is well able to separate the noise from the biologically relevant signal.

#### Biological relevance of the selected genes

We have seen that the independent principal components indicate relevant biological similarities between the samples. We next assessed whether these selected genes were relevant to the biological study. The genes selected with either sIPCA or sPCA were further investigated using the GeneGo software [26], that can output pathways, process networks, Gene Ontology (GO) processes and molecular functions.

We decided to focus only on the first two dimensions as they were sufficient to obtain a satisfying cluster of the samples (see previous results). We therefore analyzed the two lists of 50 genes selected with either sIPCA or sPCA for each of these two dimensions. Amongst these 50 genes, between 33 to 39 genes were annotated and recognized by the software.

##### Genes selected on dimension 1

Both methods selected genes previously highlighted in the literature as having functions in detoxification and redox regulation in response to oxidative stress: 2 cytochrome P450 genes (1) and heme oxygenase 1 were selected by sIPCA (sPCA) on the first dimension (see Additional files 1 and 2). The expression of these genes has been found to be altered in biological pathways perturbed subsequent to incipient toxicity [27-32]. These genes were also previously selected with other statistical approaches by other colleagues on the same study [20].

A Gene Ontology enrichment analysis for each list of genes was performed. GO terms significantly enriched included biological processes related to response to unfolded proteins, protein refolding and protein stimulus, as well as response to chemical stimulus and organic substance (Additional file 3). Although very similar, the sPCA gene list highlighted slightly more genes related to these GO terms than the sIPCA gene selection. The GO molecular functions related to these genes were, however, more enriched with sIPCA: heme and unfolded protein binding as well as oxidoreductase activity (Additional file 4).

##### Genes selected on dimension 2

The gene lists from dimension two not only highlighted response to unfolded protein and to organic substance, but also cellular carbohydrate biosynthesis process, trygliceride, acylglycerol, neutral metabolic processes as well as catabolic process and glucogenesis. For this dimension, however, it is sIPCA that selected more relevant genes that enriched these terms (Additional file 5).

In terms of pathways, both approaches selected HSP70 and HSP90 genes. The HSP90 gene encodes a member of the heat shock proteins 70 family. These proteins play a role in cell proliferation and stress response, which explained the presence of pathways found such as oxidative stress [33,34] (Additional file 6). The HSP90 proteins are highly conserved molecular chaperones that have key roles in signal transduction, protein folding and protein degradation. They play an important roles in folding newly synthesized proteins or stabilizing and refolding denatured proteins after stress [35].

##### Summary

This preliminary analysis demonstrates the ability of sIPCA and sPCA to select genes that were relevant to the biological study. These genes that are ranked as being 'important' by both approaches, participate in the determination of the components which are linear combinations of the original variables. Therefore, the expression of these selected genes not only help clustering the samples according to the different treatments or biological conditions but also have a biologically relevant meaning for the system under study.

## Conclusions

We have developed a variant of PCA called IPCA that combines the advantages of both PCA and ICA. IPCA assumes that biologically meaningful components can be obtained if most noise has been removed from the associated loading vectors. By identifying non-Gaussian loading vectors from the biological data, it better reflects the internal structure of the data compared to PCA and ICA. On simulated data sets, we showed that IPCA outperformed PCA and ICA in the super-Gaussian case, and that the kurtosis value of the loading vectors can be used to choose the number of independent principal components. On real data sets, we assessed the cluster validity using the Davies Bouldin index and showed that in high dimensional cases, IPCA could summarize the information of the data better or with a smaller number of components than PCA or ICA.

We also introduced sIPCA that allows an internal variable selection procedure. By applying a soft-thresholding penalization on the independent loading vectors, sparse loading vectors are obtained which enable variable selection. We have shown that sIPCA can correctly identify most of the important variables in a simulation study. For one data set, the genes selected with sIPCA and sPCA were further investigated to assess whether the two approaches were able to select genes that were relevant to the system under study given these genes, relevant GO terms, molecular functions and pathways where highlighted. This analysis demonstrated the ability of such approaches to unravel biologically relevant information. The expression of these selected genes is also decisive to cluster the samples according to their biological conditions.

We believe that (s)IPCA approach can be useful, not only to improve data visualization and reveal experimental characteristics, but also to identify biologically relevant variables. IPCA and sIPCA are implemented in the R package mixomics [36,37] and its associated web-interface http://mixomics.qfab.org.

## Methods

### Principal Component Analysis (PCA)

PCA is a classical dimension reduction and feature extraction tool in exploratory analysis, and has been used in a wide range of fields. There exists different ways of solving PCA. The most computationally efficient algorithm uses Singular value decomposition (SVD): suppose **X **is a centered *n *× *p *matrix (the mean of each column has been subtracted), where *n *is the number of samples (or observations) and *p *is the number of variables or biological entities that are measured. Then the SVD of data matrix **X **can be defined as

(1) $$X={\mathrm{UDV}}^{T},$$

where **U **is an *n *× *p *matrix whose columns are uncorrelated (i.e. **U^T^U = I_P_**), **V **is a *p *× *p *orthogonal matrix (i.e. **V^T^V **= **I_P_**), and **D **is a *p *× *p *diagonal matrix with diagonal elements *d_j_*. We denote **u***_j _*the columns of U and **v***_j _*the columns of **V**. Then **u***_j_d_j _*is the *jth principal component *(PC) and **v***_j _*is the corresponding *loading vector *[1]. The PCs are linear combination of the original variables and the loading vectors indicate the weights assigned to each of the variables in the linear combination. The first PC accounts for the maximal amount of the total variance. Similarly, the *jth *(*j *= 2,..., *p*) PC can explain the maximal amount of variance that is not accounted by the previous *j *- 1 PCs. Therefore, most of the information contained in **X **can be reduced to a few PCs. Plotting the PCs enable a visual representation of the samples projected in the subspace spanned by the PCs. We can expect that the samples belonging to the same biological group, or undergoing the same biological treatment would be clustered together and separated from the other groups.

#### Limitation of PCA

Sometimes, however, PCA may not be able to extract relevant information and may therefore provide meaningless principal components that do not describe experimental characteristics. The reason is that its linear transformation involves second order statistics (i.e. to obtain mutually non-orthogonal PCs) that might not be appropriate for biological data. PCA assumes that gene expression data have Gaussian signals, while it has been demonstrated that many gene expression data in fact have 'super-Gaussian' signals [2,4].

### Independent Component Analysis (ICA)

Independent Component Analysis (ICA) was first proposed by [8]. ICA can reduce the effects of noise or artefacts in the data as it aims at separating a mixture of signals into their different sources. By assuming non-Gaussian signal distribution, ICA models observations as a linear combinations of variables, or components, which are chosen to be as statistically independent as possible (i.e. the different components represent different non-overlapping information). ICA therefore involves higher-order statistics [14]. In fact, ICA attempts to recover statistically independent signal from the observations of an unknown linear mixture. Several algorithms such as FastICA, Kernel ICA [38] and ProDenICA [39] were proposed to estimate the independent components. The FastICA algorithm maximizes non-Gaussianity of each component, while Kernel ICA and ProDenICA minimize mutual information between components. In this article, we used the FastICA algorithm.

Let **X **(*n *× *p*) be the centered data matrix and **S **(*n *× *p*) the matrix containing the independent components (IC). We can solve the ICA problem by introducing a mixing matrix **A **of size *n *× *n*:

(2) X=AS.

The mixing matrix **A **indicates how the independent components of **S **are linearly combined to construct **X**. If we rearrange the equation above, we get

(3) S=WX,

where **W **(*n *× *n*) is the unmixing matrix that describes the inverse process of mixing the ICs. If we assume that **A **is a square and orthonormal matrix, then **W **is simply the transpose of **A**. In practice, it is very useful to whiten the data matrix **X**, i.e., to obtain Cov(**X**) = **I**. This allows the mixing matrix **A **to be orthogonal: Cov(**AS**) = **I **and **SS^T ^**= **I **⇒ **AA^T ^**= **I**. The orthogonality of the matrix also enables fewer parameters to be estimated. In the FastICA algorithm, PCA is used as a pre-processing step to whiten the data matrix. If we rearrange (1), we therefore obtain

(4) $$U^{T}=D^{-1}V^{T}X^{T},$$

since the columns of **V **are orthonormal. The rows of **U^T ^**are uncorrelated and have zero mean. To complete the whitening step, we can multiply **U^T ^**by $\sqrt{n-1}$, so that the rows of **U^T ^**have unit variance. Then let $\tilde{U}$ be the whitened PCs $\left(\tilde{U}=\sqrt{n-1}U^{T}\right)$. The ICs are estimated through the following equation:

(5) $$S=W\tilde{U}.$$

ICA assumes that Gaussian distribution represent noise, and therefore aims at identifying non-Gaussian components in the sample space that are as independent as possible. Recent studies have observed that the signal distribution of microarray data are typically super-Gaussian since only a small number of genes contribute heavily to a specific biological process [2,5].

Two classical quantitative measures of Gaussianity are kurtosis and negentropy.

• Kurtosis, also called the fourth-order cumulant is defined as

(6) $$K=E\left\{s_{i}^{4}\right\}-3.$$

where **s***_i _*is the row of **S**, which has zero mean and unit variance, *j *= 1... *n*. The kurtosis value equals zero if **s***_i _*has a Gaussian probability density function (pdf), is positive if **s***_i _*has a spiky pdf (super-Gaussian, i.e. the pdf is relatively large at zero) and is negative if **s***_i _*has a flat pdf (sub-Gaussian, i.e. the pdf is rather constant near zero). We are interested in the spiky and flat pdf (i.e. non-Gaussian pdfs) since non-Gaussianity is regarded as independence [9]. Note that although kurtosis is both computationally and theoretically simple, it can be very sensitive to outliers. The authors in [6] proposed to order the ICs based on their kurtosis value.

• In the FastICA algorithm, negentropy is used as it is an excellent measurement of non-Gaussianity. Negentropy equals zero if **s***_i _*is Gaussian and is positive if **s***_i _*is non-Gaussian. It is not only easy to compute, but also very robust [9]. However, this measure does not distinguish between super-Gaussianity and sub-Gaussianity.

#### Limitation of ICA

Similar to PCA, ICA also suffers from high dimensionality, which sometimes leads to the inability of the ICs to reflect the (biologically expected) internal structure of the data. Furthermore, since ICA is a stochastic algorithm, it faces the problem of convergence to local optima, leading to slightly different ICs when re-analyzing the same data [40].

### Independent Principal Component Analysis (IPCA)

To reduce noise and better reflect the internal structure of the data generated by the biological experiment, we propose a new approach called Independent Principal Component Analysis (IPCA). Rather than denoising the data or the PCs directly, as it is performed in ICA, we propose instead to reduce the noise in the loading vectors. Recall that the PCs, which are then used to visualize the samples and how they cluster together, are a linear combination of the original variables weighted by their elements in the corresponding loading vectors. Thus we will obtain denoised PCs by using ICA as a denoising process of the associated loading vectors.

**Figure 7:**
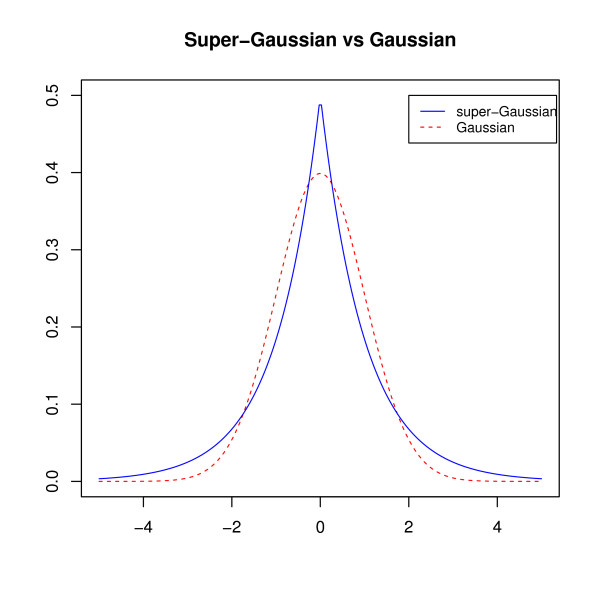
**Super-Gaussian vs. Gaussian distribution**. A super-Gaussian distribution (Laplace distribution for example) has a more spiky peak and a longer tail than a Gaussian distribution. The distribution of a noiseless loading vector is similar to a super-Gaussian distribution. If a large amount of noise exists in the loading vectors, its distribution will tend towards a Gaussian distribution.

We make the assumption that in a biological system, different variables (biological entities, such as genes and metabolites) have different levels of expression or abundance depending on the biological conditions. Therefore, only a few variables contribute to a biological process. These relevant variables should have important weights in the loading vectors while other irrelevant or noisy variables should have very small weights. In fact, once the loading vectors are denoised, we expect them to have a super-Gaussian distribution (as opposed to a Gaussian distribution when noise is included, see Figure 7 for the plot of a typical super-Gaussian and a Gaussian distribution). Maximizing non-Gaussianity of the loading vectors will thus enable to remove most of the noise. IPCA is described below and summarized in Table 6.

**Table 6:** Summary of the IPCA algorithm.

**Algorithm **Principal Component Analysis with Independent loadings (IPCA)

1. Implement SVD on the centered data matrix **X **to generate the whitened loading vectors **V**, and choose the number of components *m *to reduce the dimension.
2. Implement FastICA on the loading vectors **V **and obtain the independent loading vectors **S^T^**.
3. Project the centered data matrix **X **on the *m *independent loading vectors **s***_j _*and get the Independent PCs ${\tilde{u}}_{j},j=1...m$.
4. Order the IPCs by the kurtosis value of their corresponding independent loading vectors.

#### Extract the loading vectors from PCA

PCA is applied to the **X **(*n *× *p*) centered data matrix using SVD to extract the loading vectors:

(7) $$X=UDV^{T},$$

where the columns of **V **contain the loading vectors. Since the mean of each loading vector is very close to zero, these vectors are approximately whitened and the FastICA algorithm can be applied on the loading vectors.

#### Dimension reduction

Dimension reduction enables a clearer interpretation without the computational burden. Therefore, only a small number of loading vectors, or, equivalently, a small number of PCs is needed to summarize most of the relevant information. However, there is no globally accepted criterion on how to choose the number of PCs to keep. We have shown that the kurtosis value of the independent loading vectors gives a post hoc indication of the number of independent principal components to be chosen (see 'Results and Discussion' Section). We have experimentally observed that 2 or 3 components were sufficient to highlight meaningful characteristics of the data and to discard much of the noise or irrelevant information.

#### Apply ICA on the loading vectors

The non-Gaussianity of the loading vectors can be maximized using equation (5):

(8) $$S=W{\tilde{V}}^{T},$$

where $\tilde{V}$ is the (*p *× *m*) matrix containing the m chosen loading vectors, **W **is the (*m *× *m*) unmixing matrix and **S **is the (*m *× *p*) matrix whose rows are the independent loading vectors. The new independent principal components (IPCs) are obtained by projecting **X **on **S^T^**:

(9) $$\tilde{U}=XS^{T}$$

where $\tilde{U}$ is a (*n *× *m*) matrix whose columns contain the IPCs.

#### Ordering the IPCs

Recall that ICA provides unordered components and that the kurtosis measure indicates the Gaussian characteristic of a pdf. [6] recently proposed to use the kurtosis measure of the ICs to order them. In IPCA, we propose instead to order the IPCs according to the kurtosis value of the *m *independent loading vectors **s***_j _*(*j *= 1... *m*), as we are mainly interested loading vectors with a spiky pdf, indicated by a large kurtosis value.

### Sparse IPCA (sIPCA)

Similar to PCA and ICA, the elements in the loading vectors in IPCA indicate which variables are important or relevant to determine the principal components. Therefore, obtaining *sparse *loading vectors enables variable selection to identify important variables of potential biological relevance, as well as removing noisy variables while calculating the IPCs in the algorithm.

Various sparse PCA approaches have been proposed in the literature: SPCA [41], sPCA-rSVD [18], SPC [42]). In these approaches, the loading vectors are penalized using Lasso [43] to perform an internal variable selection. In fact, all these sparse PCA variants can be approximately solved by using soft-thresholding [17]. Our sparse IPCA therefore directly implements soft-thresholding on the independent loading vector **s***_j _*to select the variables:

(10) $${ŝ}_{jk}=sign\left(s_{jk}\right){\left(|s_{jk}|-\gamma \right)}^{+},$$

where *γ *is the threshold and is applied on each element *k *of the loading vector **s***_j _*(*k *= 1... *p, j *= 1... *m*) so as to obtain the sparse loading vector ${\hat{s}}_{j}$. The variables whose original weights are smaller than the threshold *γ *will be penalized to have zero weights. A classical method to choose *γ *is cross-validation. In practice, however, *γ *has been replaced by the degree of sparsity (i.e., the number of non-zero elements in each loading vector, see following paragraph). In this way, we can control how many variables to select and save some computational time.

### Using (s)IPCA

IPCA and sIPCA are implemented in the R package mixomics which is dedicated to the analysis of large biological data sets [36,37]. The use of the approaches is straightforward: the user needs to input the data set, and to choose the number of components to keep (usually set to a small value). In the case of the sparse version, the number of variables to select on each sIPCA dimension must also be given. The number of components can be reconsidered afterwards by extracting the kurtosis value of the loading vectors, i.e., identifying when a sudden drop occurs in the obtained values will indicate how many components are enough to explain most of the information in the data.

The number of variables to select is still an open issue (as pinpointed by many authors working on sparse approaches, [18]) as in such studies, we are often limited by the number of samples. Tuning the number of variables to select therefore mostly relies on the biological question. Sometimes, an optimal but too short gene selection may not suffice to give a comprehensive biological interpretation, and sometimes, the experimental validation might be limited in the case of a too large gene selection.

In our example, for the sake of simplicity, we have set the same number of variables to select on each dimension.

### Simulation studies

In the different simulation studies, we used the following framework (previously proposed by [18]). **Σ **is the variance-covariance matrix of size 500 × 500, whose first two normalized eigenvectors **v**_1 _and **v**_2_, both of length 500 are simulated for different cases described the the 'Results and Discussion' Section. The other eigenvectors were drawn from *U*0[1]. A Gram-Schmidt orthogonalization method was applied to obtain the orthogonal matrix **V **whose columns contain **v**_1 _and **v**_2 _and the other eigenvectors. To make the first two eigenvectors dominate, the first two eigenvalues were set to *c*_1 _= 400, *c*_2 _= 300 and *c_k _*= 1 for *k *= 3,..., 500. Let **C **= *diag*{*c*_1_,..b., *c*_500_} the eigenvalue matrix, then **Σ **= **VCV^T^**. The data are then generated from a multivariate normal distribution N(**0**, **Σ**), with *n *= 50 samples and *p *= 500 variables.

### Davies-Bouldin index

Davies-Bouldin measure is an index of crisp cluster validity [19]. This index compares the within-cluster scatter with the between-cluster separation. It was chosen in this study because of its statistics and geometric rationale. The Davies-Bouldin index is defined as

$$\frac{1}{K}\overset{K}{\underset{i=1}{\sum }}\underset{i\neq j}{\text{max}}\frac{\sigma_{i}+\sigma_{j}}{d\left(c_{i},c_{j}\right)},$$

where *c_i _*is the centroid of cluster *i*, and *σ_i _*is the average distance of all elements in cluster *i *to centroid *c_i _*and *d*(*c_i_, c_j_*) is the distance between the two centroids, *K *is the number of known biological conditions or treatments. Depending on the number of components that were chosen, we applied a 2- or 3-norm distance. Geometrically speaking, we are seeking to minimize the within-cluster scatter (the numerator) while maximizing the between class separation (the denominator). Therefore, for a given number of components, the approach that gives the lowest index has the best clustering ability.

## Competing interests

The authors declare that they have no competing interests.

## Authors' contributions

FY performed the statistical analysis, wrote the R functions and drafted the manuscript. KALC participated in the design of the manuscript and helped drafting the manuscript. JC participated in the implementation of the R functions and implemented IPCA in the web-interface. All authors read and approved the final manuscript.

## Supplementary Material

Additional file 1 **List of genes from sIPCA**. List of genes and gene title selected by sIPCA on each dimension on Liver Toxicity study. Click here for file

Additional file 2 **List of genes from sPCA**. List of genes and gene title selected by sPCA on each dimension on Liver Toxicity study. Click here for file

Additional file 3 **GeneGo analysis**. Comparison of the GO processes for the genes selected on dimension 1 with sIPCA and sPCA on Liver Toxicity study. Click here for file

Additional file 4 **GeneGo analysis**. Comparison of the GO molecular functions for the genes selected on dimension 1 with sIPCA and sPCA on Liver Toxicity study. Click here for file

Additional file 5 **GeneGo analysis**. Comparison of the GO processes for the genes selected on dimension 2 with sIPCA and sPCA on Liver Toxicity study. Click here for file

Additional file 6 **GeneGo analysis**. Comparison of the GeneGO pathways maps for the genes selected on dimension 1 with sIPCA and sPCA on Liver Toxicity study. Click here for file
